# Epidemiological study of critical burn patients in an ICU

**DOI:** 10.1186/cc11070

**Published:** 2012-03-20

**Authors:** L Cachafeiro, M Sanchez, E Herrero, J Camacho, M Hernandez, A Agrifoglio, A García de Lorenzo, M Jimenez

**Affiliations:** 1 Hospital La Paz, Madrid, Spain

## Introduction

Burn injuries remain a significant problem with high associated morbidity and mortality, long average stays and high costs. The aim of our study is to analyze the epidemiology and mortality of critical burn patients admitted to the ICU at a university hospital in Madrid, Spain.

## Methods

We performed a prospective, observational and descriptive study in patients admitted with burns over 20% of the total body surface area (TBSA), from October 2008 to December 2009. Demographic data were collected, TBSA, location and mechanism of burns, severity scores (ABSI, APACHE II, SOFA at admission, and next 3 days) length of stay, complications and mortality. Data are presented as number and percentage or as median and interquartile range, and they were analyzed with the Fisher exact test and Mann-Whitney test.

## Results

**Figure 1:**
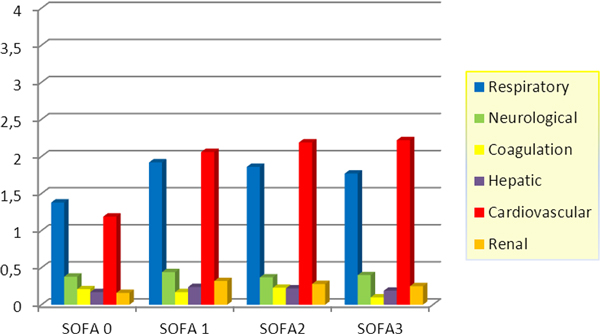


During this period, 64 patients were admitted to our unit, 45 (70.3%) were men and 19 (29.7%) were women. The mean age was 48 ± 19. SOFA score at admission was 3 ± 2, APACHE II score 15 ± 6 (range 4 to 38) and ABSI 8 (range 5 to 16). The TBSA average was 40 ± 20% and the mechanism of burn was by flame in 60 patients (93.8%), scald in four (6.3%), electrical in two (3.1%) and chemical in one (1.6%). The most frequent location was in the upper limbs in 60 patients (93.8%), followed by thorax in 50 (78.19%), head and neck in 43 (67.2%), lower limbs in 43 (67.2%), and back in 29 (45.3%). Six patients had trauma associated and 23 had inhalation injury. Thirty-two patients (50.0%) required escharotomy at admission and 16 (25.0%) had compartment syndrome. Forty-four patients (68.8%) needed mechanical ventilation, and 20 (31.3%) tracheostomy. Fifty-six patients had complications. The most frequent were: shock (70.3%), ARDS (31.3%), sepsis (35.9%) and renal failure (26.6%). All complications increased significantly the mortality (*P *< 0.001). The length of stay was 30 days and global mortality was 29.7% (19 patients). See Figure 1.

## Conclusion

In our study the most common burns were caused by flame in the upper limbs, chest, neck and face. Eighty-nine percent of our patients had complications, and they increased significantly the length of stay and mortality. Based on the SOFA score, patients had higher scores for respiratory and cardiovascular systems. However, mortality was lower than expected in severity scores.

